# The Impact of Crisis Characteristics and Media Coverage on the Public’s Attitude Toward Tourism Organization Expressed on Sina Weibo

**DOI:** 10.1007/978-3-030-65785-7_28

**Published:** 2020-11-28

**Authors:** Lijuan Su, Svetlana Stepchenkova

**Affiliations:** 1 Department for Informatics, Technical University of Munich, Garching bei München, Bayern Germany; 2 Smart Tourism Education Platform (STEP) College of Hotel and Tourism Management, Kyung Hee University, Seoul, Korea (Republic of); 3 Department of Tourism and Service Management, MODUL University Vienna, Vienna, Wien Austria; 4 Sun Yat-Sen University, Zhuhai, 519082 China; 5 University of Florida, Gainesville, FL 32611 USA

**Keywords:** Crisis communication, Machine-learning, Response strategies, Sina Weibo, Social media, Tourism and hospitality

## Abstract

Tourism and hospitality crises that are extensively discussed online are damaging to organizational image and reputation; therefore, choosing effective response strategies is of paramount importance for service providers. The online discussions data from six hospitality and tourism related crises were used to test which crisis and media coverage characteristics significantly affected the public’s emotional and behavioral reactions to crises. With reference to the attribution theory and the situational crisis communication theory, this study identified the potentially influential crisis characteristics, hypothesized their relationship with variables describing consumer reactions to crises, and then tested those relationships in a series of ANOVA and hierarchical regression analyses. Results indicated that the locus of control, crisis stability, attribution of organizational responsibility, and organizational response strategy affected the public’s cognitive and emotional responses to crises most strongly. The attractiveness and goodwill of media sources also had an effect, as well as the quality and fairness of messages. This study makes a methodological contribution to tourism research by training machine-learning classifiers prior to conducting hypothesis testing. Identifying the most influential factors affecting the public’s response to crises can serve as guidelines for tourism and hospitality organizations in monitoring the spread of online crisis discussions and developing the most appropriate response in order to minimize consumers’ negative emotions that affect online and off-line behavior toward the organization and its brand.

## Introduction

As any industry, tourism and hospitality have experienced their share of large-scale disasters, and small-scale crises that have been widely disseminated on social media. The disaster emergency management research has mostly dealt with the impact of a crisis at the national and regional levels and looked at the effects of those disasters on the whole business sectors, primarily from the regional governing and planning perspective (e.g., Reuter and Kaufhold 2018; Ritchie and Jiang 2019). In the hospitality industry, however, a large share of crises is originated in the service failure incidents which the public take online where they are quickly and broadly disseminated. Limited research has examined the impact of such crises on the involved organizations and how tourism and hospitality companies could process online crisis information and effectively communicate their response (e.g., Ritchie and Jiang 2019; Lin et al. 2019).

In the digital era, social media provide a multitude of real-time interactive platforms that are actively used by organizations for their brand-building and marketing efforts as well as for crisis communications. Crisis communications, however, mainly include warnings about coming disasters (e.g., floods, hurricanes, or fires), providing evacuation guidelines, and dispensing advice for optimal behavior in emergencies (e.g., Reuter et al. 2016). It has been noted that most commercial organizations are reluctant to use social media for direct communications with the public about service failure crises of high visibility (Barbe and Pennington-Gray 2018; Su et al. 2019). Organizations, including tourism and hospitality enterprises, are not certain whether those communications would improve the public image of the company or create more tensions between the company and their current and potential customer base.

In the absence of solid research on the subject, the guidelines are vague and the selected strategies are largely “trial and error” (Eriksson and Olsson 2016). Three different response strategies are mainly adopted: no response, the accommodative, and defensive strategies. The accommodative strategies refer to responses that “accepts responsibility, admits to the existence of problems”; the defensive strategies refer to responses that “insists that the problems do not exist, tries to alleviate doubts about the firm’s ability to generate future revenue” (Marcus and Goodman 1991).

This study examined the social media coverage of six tourism crises that were widely disseminated on China’s largest social media platform Sina Weibo. The purpose of the study was to identify which crisis characteristics, especially the organizational response strategies types, affected the public’s levels of information processing and attitudes, that is, acceptance and rejection of organizational response, boycotts, online reposting, and emotions, the most. The choice of crisis characteristics was guided by the attribution theory (Weiner 1986) and the situational crisis communication theory (Coombs 2007), which indicated that the crisis attribution, organizational responsibility, crisis response strategies had impacts on customers’ emotions and online behaviors. The study also considered the impact of crisis media coverage.

## Research Context

Brief descriptions of the selected crises are provided below. The crises were classified with respect to crisis attribution type, responsibility attribution, organizational response strategies, and the credibility of the media source and message.

### 2018 Five-Star Hotels’ Hygiene Horror (HHH).

A Chinese whistle-blower posted a 12-min video on Weibo, recording how housekeepers violated hygiene protocols in 14 luxury hotels in November 2018. The posting triggered nearly 1.6 million comments. The hotels adopted various response strategies (e.g., scapegoating, denial).

### 2017 Five-Star Hotel Unchanged Bedsheet.

On September 4, 2017, Lanmei Test released a video that provided evidence that five inspected five-star hotels in Beijing did not change bedsheets for the next guest. The involved hotels used the no response or denial strategy after the video was widely disseminated online (China Daily, 2017).

### 2017 Haidilao Hotpot Chain Rat Infest Scandal.

On August 25, 2017, a journalist posted a video showing rats infesting the kitchen in two branches of a popular Sichuan-based hot pot chain Haidilao in Beijing. Haidilao admitted their fault and apologized online within three hours of the video posting, and followed by closing the branches.

### 2019 Taste of Grandma Restaurant Hygienic Scandal.

On March 15, 2019, the hygiene problems in two branches of a famous Chinese restaurant chain, Taste of Grandma, were exposed. The company apologized and further invited a third-party company to conduct inspections for all its branches.

### 2019 Boycott to Cathay Pacific on Sina Weibo.

In August of 2019, Cathay Pacific received an official warning from the Civil Aviation Administration of China regarding violating aviation rules. #Boycott to Cathay Pacific got 51 million views on Weibo.

### 2020 OTAs and Airlines Refund Policies to Covid-19.

Facing the outbreak of Covid-19, Chinese online travel agencies faced a high volume of canceled trips. The number of customer complaints on Tujia, Ctrip, and Zhixing increased by 1353%, 427%, and 142% in January, respectively, compared to December of 2019 (Blackcat 2020).

## Method

A total of 254,206 comments under 638 most popular posts about six selected crises were collected, and 216,288 comment messages were cleaned for analysis. Using the HHH crisis dataset, the optimal feature parameters and classifiers were selected among five supervised algorithms. The bagging tree (Predictor subset = 83, Interaction depth = 12) had the lowest cross-validated error rate, as well as the highest accuracy, recall, and F-score to categorize emotions (Table 1). The bagging algorithm was used on the full dataset.

**Table 1.** Model performance of five supervised algorithms.

Method	Train.err	Test.err	CV.err	Accuracy	Precision	Recall	F
KNN	.4852	.6982	.6761	.5648	.1999	.2540	.2237
Bagging	.0138	.4751	.5237	.5953	.3007	.3819	.3365
Boosting	.2867	.4908	.5405	.5919	.3800	.2840	.3251
SVM	.0414	.4803	.5847	.5831	.2870	.2842	.2856
Naïve Bayes	.9213	.9053	.9174	.5165	.2201	.1257	.1600

Next, 11,600 (5.36%) messages were randomly selected from the full dataset to manually label the public’s levels of (1) information processing, (2) boycotting and buying intentions, (3) attitudes to response strategies, (4) reposting intentions, and (5) emotion types. For example, the levels of information processing involved attention, comprehension, elaboration, and not mentioned. Two coders were recruited to manual label the dataset independently and agreement was achieved for the inconsistent results.

Five automated classifiers were trained with the bagging algorithm to categorize the above variables based on the TF-IDF matrix of the tokens extracted from the texts. The K-fold cross-validation (K = 5) procedure was employed to measure the model performance. The out-of-bag errors were 22.33%, 16.66%, 11.59%, 0.88%, and 58.87%, respectively. The high OOB of emotions was related to the number of emotional types, including seven categories (e.g., anger, contempt, sadness).

## Findings

### Differences in Three Types of Crisis Response Strategies

A series of ANOVA analyses were conducted to examine how the general public reacted to crises depending on the organizational response strategy. The no response strategy outperformed another two strategies, with significantly lower levels of information processing, rejection of organizational response, and anger. The defensive strategy had significantly larger scores in the anger ratio and rejection ratio; while the last one had higher scores of the information processing, boycotts, and rejections.

### Impact on Levels of Information Processing

The study utilized hierarchical regression models to predict the public’s average level of information processing (Table 2). Dummy variables were created for categorical variables.Three crisis attributions, organizational responsibility, and crisis response strategies were input in step one and explained 22.17% of the total variances (R2 = .2492, Adj R2 = .2217, F = 9.06). The internal locus of crisis and the defensive strategies had significant impacts, while stability was marginally significant. Posts with defense strategies resulted in a higher average level of information processing. After the entry of credibility variables, no significant improvement was detected (ΔF = .7168).

**Table 2.**
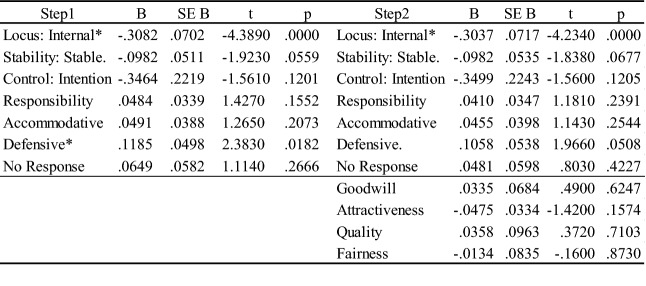
Regression results in the average scores of information processing.

### Impacts on Rejection and Acceptance to Crisis Response Strategies

The frequency of messages mentioning the rejection of organizational crisis strategies was predicted by both crisis attribution and credibility variables, as well as the goodwill and attractiveness of media sources. The accommodative strategies had significantly positive impact on these variances (t = 3.459, p = .0007). In the model to predict the rejection ratio, only the crisis attribution and response strategies had impacts.

As for the acceptance to crisis response strategies, accommodative strategies (t = 3.027, p = .0028) and organizational responsibility (t = 1.661, p = .0980) had significantly and marginally positive effects. The accommodative strategies also had a significant positive impact to predict the acceptance ratio (t = 5.255, p < .001).

### Impacts on Anger

SCCT model indicated that both the organizational responsibility and crisis response strategies had an impact on emotions, especially the emotion of anger. For the frequency of anger, the internal locus had a significantly negative impact, while organizational responsibility, accommodative strategies, and defensive strategies had a significantly positive impact. However, the entry of credibility variables failed to improve the prediction model significantly (F = 1.4443, p = .2211).

For the ratio of anger, the basic model explained 37% of the total variances. The internal locus (t = −3.870, p < .001) and the stable attribution (t = −2.750, p = .0065) had a significantly negative impact; organizational responsibility (t = 3.517, p < .001), accommodative strategies (t = 3.871, p < .001), and defensive strategies (t = 6.809, p < . 001) had significantly positive effects. The entry of credibility variables could improve the model significantly (F = 2.7536, p = .0294).

## Discussion and Conclusion

Crisis characteristics depicted in the popular posts could successfully predict the public’s levels of information processing, attitudes to the crisis response strategies, and the emotion of anger retrieved from the comments and replies, but could not predict the publics’ calls for boycotts and reposting behaviors. This study indicates that crises with the internal and stable attribution result in more negative responses towards the involved organization. The involved organization would be suggested to take no responses. For another two strategies, PR could make a decision purposefully: the defensive strategies lead to a higher ratio of rejections to responses, boycotts, and anger; while the accommodative strategies lead to a higher volume of online discussions.
